# Intrathecal Trastuzumab for HER2-Positive Cancer of Unknown Primary Leptomeningeal Metastasis: A Case Report

**DOI:** 10.7759/cureus.57322

**Published:** 2024-03-31

**Authors:** Kohei Oka, Shun Futamura, Taishi Harada

**Affiliations:** 1 Department of Medical Oncology, Fukuchiyama City Hospital, Fukuchiyama, JPN

**Keywords:** leptomeningeal metastasis, her2-positive, intrathecal chemotherapy, chemotherapy, cancer of unknown primary

## Abstract

Cancer of unknown primary (CUP) and leptomeningeal metastasis are difficult conditions with limited treatment options. We report a case of CUP leptomeningeal metastasis that was refractory to empirical chemotherapy but achieved a favorable response to intrathecal trastuzumab after the identification of human epidermal growth factor receptor-2 (HER2) amplification. A 59-year-old woman was diagnosed with CUP with metastasis of a poorly differentiated carcinoma to the left axillary, anterior mediastinal, peritoneal, and bilateral supraclavicular lymph nodes. Leptomeningeal metastasis was confirmed shortly after she started empiric chemotherapy; empiric therapy with intrathecal methotrexate failed to relieve her symptoms. Meanwhile, the lymph node specimen tested positive for HER2 amplification. She underwent intrathecal trastuzumab, then her neurological symptoms resolved the following day. We suggest that intrathecal trastuzumab is an effective treatment for HER2-positive CUP leptomeningeal metastasis.

## Introduction

Leptomeningeal metastasis, also known as leptomeningeal carcinomatosis, is a fatal manifestation of metastatic carcinoma with limited treatment options [1]. It frequently presents with neurological symptoms, including headaches, confusion, and seizures [2]. Its treatment options are limited because the blood-brain barrier restricts the penetration of chemotherapeutic agents into cerebrospinal fluid (CSF) [3]. Intrathecal methotrexate (MTX) remains a common treatment for leptomeningeal metastasis, despite the unfavorable prognosis and adverse events [4].

Cancer of unknown primary (CUP), defined as a histologically confirmed metastatic tumor with no identifiable primary site, also presents a poor prognosis, with limited therapeutic options [5]. Systemic chemotherapy combining taxane and platinum is a standard therapy for patients with CUP when the primary site cannot be estimated, however, its efficacy is limited [5]. Herein, we report a case of leptomeningeal metastasis in a patient with human epidermal growth factor receptor-2 (HER2) positive CUP treated with intrathecal trastuzumab, a monoclonal anti-HER2 antibody.

## Case presentation

**Figure 1:**
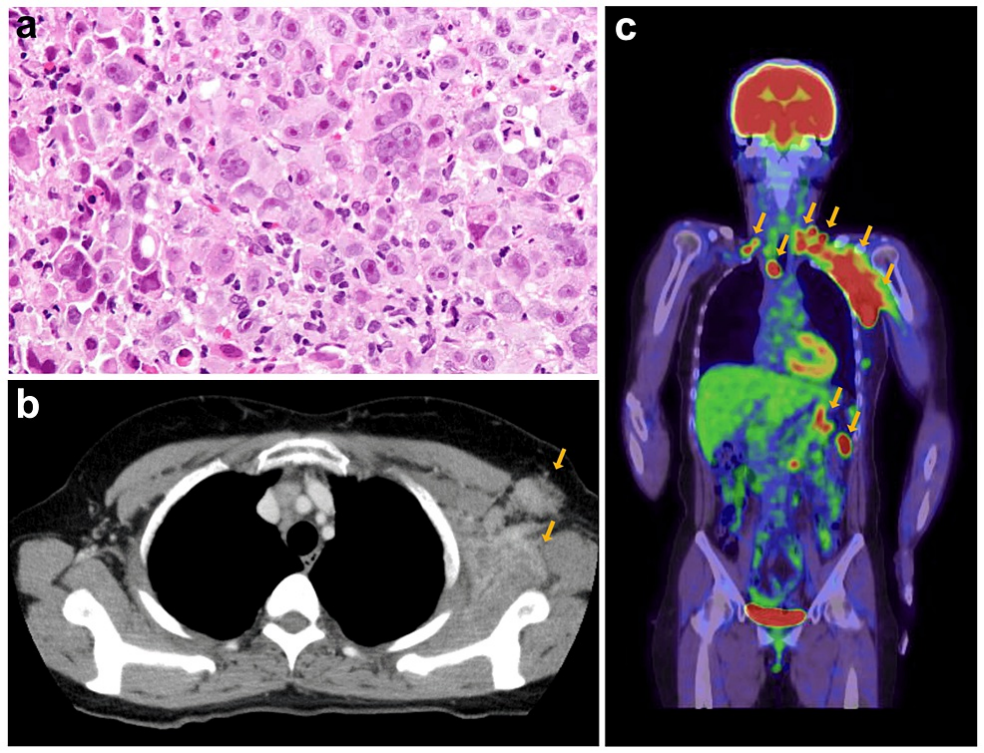
Histopathology of the biopsy specimen and CT/PET CT scan images (a) Histopathological examination of the core needle biopsy specimen for the axillary mass. Hematoxylin and eosin staining shows poorly differentiated carcinoma (magnification 40×). (b) CT scan shows the left axillary node swelling (arrows). (c) FDG-PET CT scan shows metastasis to the left axillary, anterior mediastinal, peritoneal, and bilateral supraclavicular lymph nodes (arrows). CT, Computed tomography; FDG-PET, Fluorodeoxyglucose-positron emission tomography

A 59-year-old Japanese woman presented to our hospital with left supraclavicular and axillary masses. A core needle biopsy of the axillary mass revealed a poorly differentiated carcinoma (Figure 1a). Whole-body contrast-enhanced computed tomography (CT) and fluorodeoxyglucose-positron emission tomography (PET)-CT scans indicated metastasis to the left axillary, anterior mediastinal, peritoneal, and bilateral supraclavicular lymph nodes (Figures 1b, 1c). Serum tumor marker analysis showed elevated levels of cytokeratin 19 fragment (CYFRA), carcinoembryonic antigen (CEA), carbohydrate antigen 15-3 (CA15-3), and National Cancer Center-Stomach-439 (NCC-ST-439) (Table 1).

**Table 1:** Serum tumor markers at cancer diagnosis HCG, human chorionic gonadotropin; CYFRA, cytokeratin 19 fragment; SCC, squamous cell carcinoma; NSE, neuron-specific enolase; CEA, carcinoembryonic antigen; CA15-3, carbohydrate antigen 15-3; CA125, carbohydrate antigen 125; AFP, alpha-fetoprotein; NCC-ST-439, National Cancer Center-Stomach-439

Tumor markers	Value	Reference range
HCG (mIU/mL)	0.6	≤3.0
CYFRA (ng/mL)	17.0	≤3.5
SCC (ng/mL)	0.5	≤0.5
NSE (ng/mL)	14.7	≤16.3
CEA (ng/mL)	>100	≤5.0
CA15-3 (U/mL)	211.3	<27.0
CA125 (U/mL)	16.0	≤35.0
AFP (ng/mL)	5.0	≤10.0
NCC-ST-439 (U/mL)	3054	<4.5

Immunohistochemical staining showed that the tumor cells were positive for cytokeratin (CK) AE1/AE3, CK7, and CK34βE12; partially positive for CK20; and negative for CK5/6, p40, TTF-1, Napsin-A, LCA, S-100, ER, and PgR. However, these analyses did not identify the specific primary site.

**Figure 2:**
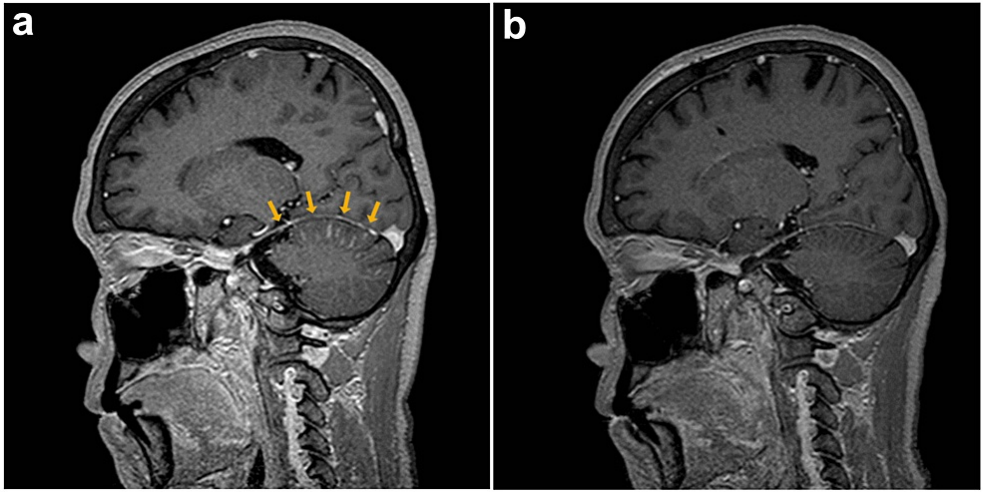
Contrast-enhanced MRI of the brain (a) Contrast-enhanced MRI of the brain at the diagnosis of leptomeningeal metastasis shows leptomeningeal enhancement around the cerebellum (yellow arrows). (b) Contrast-enhanced MRI one month after the initiation of intrathecal trastuzumab shows a decrease in enhancement around the cerebellum. MRI; magnetic resonance imaging

Thereafter, the patient underwent empiric systemic chemotherapy with carboplatin and nano-particle albumin-bound paclitaxel under the diagnosis of CUP, with simultaneous palliative radiotherapy for the left axillary and supraclavicular lymph nodes (45Gy/15fr.); however, she developed headache and dizziness during the first course of chemotherapy. Contrast-enhanced MRI of the brain showed leptomeningeal enhancement around the cerebellum (Figure 2a). Lumbar puncture revealed a poorly differentiated carcinoma in the CSF. Therefore, the patient was diagnosed with leptomeningeal metastasis of CUP. She began empiric therapy with intrathecal MTX (15 mg, weekly) for leptomeningeal metastasis; however, her symptoms persisted. Additional histopathological examination of the core needle biopsy specimen revealed the metastasis was positive for HER2 amplification: IHC 2+ and fluorescence in situ hybridization (FISH). Therefore, she received intrathecal trastuzumab (60 mg, weekly) for suspected HER2-positive breast cancer leptomeningeal metastasis. The patient’s neurological symptoms resolved the following day without any adverse effects.

**Figure 3:**
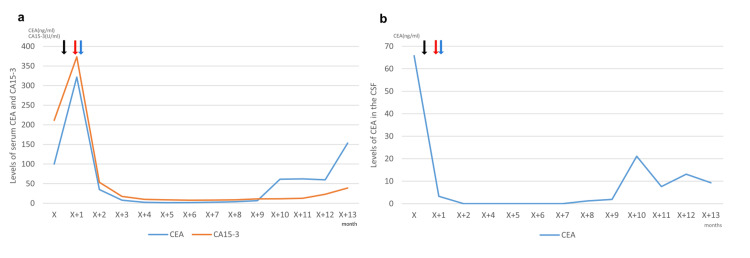
The levels of serum CEA and CA15-3 and the levels of CEA in the CSF (a) The levels of serum CEA and CA15-3. (b) The levels of CEA in the CSF. The black arrow indicates the first administration of intravenous carboplatin and nano-particle albumin-bound paclitaxel, red arrow indicates that of intrathecal trastuzumab, and blue arrow indicates that of intravenous paclitaxel, trastuzumab and pertuzumab. “X” in X-axis indicates the date of cancer diagnosis. CEA, carcinoembryonic antigen; CA15-3, carbohydrate antigen 15-3; CSF, cerebrospinal fluid

Thereafter, the patient underwent systemic therapy with paclitaxel, trastuzumab, and pertuzumab with concomitant intrathecal chemotherapy. One month later, a contrast-enhanced MRI showed a decrease in leptomeningeal enhancement around the cerebellum (Figure 2b). Two months later, she discontinued intrathecal MTX because of MTX-related leukoencephalopathy. The level of CEA in the CSF decreased immediately after the initial administration of intrathecal trastuzumab, while the levels of serum CEA and CA15-3 decreased gradually and were controlled for more than half a year (Figures 3a, 3b). The CSF cytology showed the disappearance of cancer cells 2 months after the initial administration of intrathecal trastuzumab, although they reappeared later. Intrathecal trastuzumab was continued for 12 months until contrast-enhanced MRI suggested the progression of the meningeal dissemination.

## Discussion

Leptomeningeal metastasis is a condition with progressive neurological symptoms and a poor prognosis of three to four months [1]. CUP also has a poor prognosis, except in patients with favorable subsets [5]. In patients with favorable subsets in which the primary site can be estimated, it is recommended that they be treated similarly to patients with cancers of an equivalent known primary site with metastatic dissemination. According to the National Comprehensive Cancer Network (NCCN) and European Society for Medical Oncology (ESMO) clinical practice guidelines, female patients with isolated axillary lymph node metastasis of unknown origin are recommended to be treated for breast cancer [6]. The NCCN guidelines also suggest that carcinomas of the mediastinal and supraclavicular nodes are also indicative of primary breast cancer.

Trastuzumab, an essential drug for HER2-positive breast cancer [7], is unable to penetrate the blood-brain barrier due to its high molecular weight (145 kDa) [8]; therefore, intrathecal administration of trastuzumab is anticipated to enhance bioavailability within the CSF. Several case reports have addressed the efficacy of intrathecal trastuzumab administration in patients with HER2-positive breast cancer leptomeningeal carcinomatosis [9-15]. Furthermore, a phase II study reported clinical neurological responses to intrathecal trastuzumab in this subset of patients [16]. According to the phase II study, headache, seizure, and allergic reaction were reported as adverse effects possibly related to intrathecal trastuzumab. To the best of our knowledge, this is the first case report on intrathecal trastuzumab treatment for HER2-positive CUP leptomeningeal carcinomatosis.

Although the distribution of lymph node metastasis appears to suggest that the breast is the primary site, we could not identify the primary lesion using mammography, breast MRI, or ultrasound. In addition, we could not obtain comprehensive results for tumor markers before the IHC assay was reported. The patient was refractory to empiric therapy. If the primary site could not be estimated, the patient’s neurological symptoms would not be relieved.

It is possible that this result applies only to patients with CUP in whom the breast is suspected to be the primary site. A previous report described that overexpression of the HER2 protein occurred in 11% of poorly differentiated CUP [17]. However, it remains unclear whether intrathecal trastuzumab is effective in patients with HER2-positive CUP whose primary site is not indicated.

## Conclusions

In summary, we encountered a case of HER2-positive CUP leptomeningeal metastasis. Leptomeningeal metastasis poses significant challenges due to its limited treatment options; therefore, we should continue to make an effort to estimate the primary site even after initiating empiric therapy for CUP. We suggest that intrathecal trastuzumab is an effective treatment for HER2-positive CUP leptomeningeal metastasis when the breast is considered the primary site.
